# Editorial: Nutrition, Immunity, and Lung Health: Time to Take Center Stage

**DOI:** 10.3389/fnut.2021.797554

**Published:** 2021-11-23

**Authors:** Emma J. Derbyshire, Philip C. Calder

**Affiliations:** ^1^Nutritional Insight, London, United Kingdom; ^2^School of Human Development and Health, Faculty of Medicine, University of Southampton, Southampton, United Kingdom; ^3^National Institute for Health Research Southampton Biomedical Research Centre, University Hospital Southampton National Health Service (NHS) Foundation Trust and University of Southampton, Southampton, United Kingdom

**Keywords:** antibiotic resistance, COVID-19, immunity, lung health, nutrition, respiratory tract infections, SARS-CoV-2, vitamin D

Globally, we are increasingly becoming subject to a torrent of factors that are taking their toll on lung health. Antibiotic resistance, the emergence of Severe Acute Respiratory Syndrome-2 (SARS-CoV-2) and air pollution are just some of the main drivers behind poor lung health in the modern world. In the past, there has been a generic tendency to focus on other disease entities (Alzheimer's disease, cardiovascular disease, cancer, diabetes, and stroke) and research related to lung health has lagged behind (1).

Chronic respiratory diseases have been overlooked as noncommunicable diseases, yet are one the greatest killers of present day. Chronic obstructive pulmonary disease (COPD) was the third largest global cause of death in 2019 and lower respiratory infections the fourth (2). It is only now, in the era of the coronavirus-19 (COVID-19) pandemic, that an explosion of new research coined the “infodemic” has rapidly shifted the focus towards lung health. This special issue and e-book provides novel input into the field.

In this issue, there are eight papers focusing specifically on nutrition, immunity, and lung health. There has been an emergence of evidence of the immunomodulatory roles of nutrients that could influence respiratory disease risk and progression and their possibilities as adjunctives to conventional treatment regimens. For example, Gozzi-Silva concluded that a range of vitamins (A, C, D, and E), minerals (iron, selenium, magnesium, and zinc), flavonoids, fatty acids, and certain other bioactive compounds have potential roles in reducing the risk of chronic pulmonary diseases and viral infections, due to their anti-inflammatory and antioxidant effects and ability to promote immune responses against pathogens. Singh et al. focused on evidence for nutraceuticals (from human and laboratory studies), concluding that certain vitamins (A, B, C, D, and E), probiotics, bioactive compounds (curcumin, epigallocatechin gallate, resveratrol, and quercetin) and functional foods providing these (e.g., berries and honey) could be beneficial for the immune system but should not be a replacement for a healthy lifestyle when used in supplement form.

Fernández-Lázaro et al. collated evidence on the role(s) of glucans, with particular focus on the protein/polysaccharide AM3, a natural glycophosphopeptical. This has previously been found to modulate the progression of respiratory diseases by regulating innate and adaptive immunity (altering natural killer cell production and interferon secretion and reducing inflammatory cytokine production). Whilst experimental models suggest some promise, clinical trials of these agents are now needed.

Derbyshire and Calder contributed two articles. The first, “Respiratory Tract Infections and Antibiotic Resistance”, focused specifically on the role(s) of vitamin D from a “prehabilitation” stance discussing evidence in relation to how appropriate supplementation could lower the risk of acute respiratory tract infections (ARTIs) and, in turn, play a central role in helping to reduce over-reliance on antibiotics which is contributing to antimicrobial resistance (AMR) (Derbyshire and Calder)—one of the largest pending threats to global health. The second article, “Bronchiectasis—Could Immunonutrition Heave a Role to Play in Future Management?” focused specifically on the condition bronchiectasis—a condition rising in prevalence where the bronchial tubes become permanently widened thus predisposing the lungs to infections. Research is emerging studying the roles of malnutrition and certain nutrients—vitamin D and zinc—and the lung microbiome, but future research is needed to drive advancements in this field forward (Derbyshire and Calder).

Sun et al. discussed the role(s) of gut microbiota in pulmonary disease acting *via* the gut-lung axis. Gentamicin (a broad-spectrum antibiotic) was found to disrupt the gut microbiota which could contribute to enhanced severity of influenza viral infection. Chen et al. from King's College London investigated Vitamin D mechanisms in airway diseases. *In vitro* and *in vivo* work showed that vitamin D appears to increase alpha-1 antitrypsin synthesis by human T cells, and suggests that alpha-1 antitrypsin could represent an intermediate player in some of the immunomodulatory functions of vitamin D. Concentrating on a murine model of tuberculosis (TB). Hayford et al. concluded that omega-3 polyunsaturated fatty acid therapy alongside conventional TB medications could improve anemia of infection and lower cytokine-mediated inflammation. Human studies are now needed to extend these findings.

To conclude, the SARs-CoV-2 pandemic has emphasized the fact that research into chronic respiratory/lung diseases warrants more attention. Whilst it is appreciated that we are living in a world of competing public health priorities, it is now time for lung health to take far greater precedence. Acting directly *via* immunomodulatory effects and indirectly *via* the microbiota, nutrition has a central role to play in preventative healthcare. Just as a Mediterranean diet is advocated for heart health (3), we should now begin to delve deeper into what is warranted to sustain lung health.

## Author Contributions

ED wrote the first version. PC edited and contributed to this yielding a second version. All authors contributed to the article and authorized the submitted version.

## Conflict of Interest

ED is the Director of Nutritional Insight. The remaining author declares that the research was conducted in the absence of any commercial or financial relationships that could be construed as a potential conflict of interest.

## Publisher's Note

All claims expressed in this article are solely those of the authors and do not necessarily represent those of their affiliated organizations, or those of the publisher, the editors and the reviewers. Any product that may be evaluated in this article, or claim that may be made by its manufacturer, is not guaranteed or endorsed by the publisher.
